# Influence of additional weight on the frequency of kicks in infants with Down syndrome and infants with typical development

**DOI:** 10.1590/bjpt-rbf.2014.0029

**Published:** 2014

**Authors:** Gabriela L. Santos, Thaís B. Bueno, Eloisa Tudella, Jadiane Dionisio

**Affiliations:** 1 Department of Physical Therapy, Universidade Federal de São Carlos (UFSCar), São Carlos, SP, Brazil; 2 School of Physical Education and Physical Therapy (FaeFi), Universidade Federal de Uberlândia (UFU), Uberlândia, MG, Brazil

**Keywords:** early intervention, child development, rehabilitation

## Abstract

**BACKGROUND:**

Infants with Down syndrome present with organic and neurological changes that may lead to a delay in the acquisition of motor skills such as kicking, a fundamental skill that is a precursor of gait and is influenced by intrinsic and extrinsic factors. Therefore, this movement should be taken into account in early physical therapy interventions in infants.

**OBJECTIVE:**

To analyze and to compare the effect of additional weight on the frequency of kicks in infants with Down syndrome and infants with typical development at 3 and 4 months of age.

**METHOD:**

Five infants with Down syndrome and five with typical development at 3 and 4 months of age were filmed. The experiment was divided into four experimental conditions lasting 1 minute each: training, baseline, weight (addition of ankle weight with 1/3 the weight of the lower limb), and post-weight.

**RESULTS:**

There were significant differences between groups for all variables (p<0.05), with lower frequencies observed for infants with Down syndrome in all variables. There were significant differences between the experimental conditions baseline and post-weight (p<0.001) for both groups in the frequency of contact and success, with a higher frequency in the post-weight condition.

**CONCLUSIONS:**

The weight acted as an important stimulus for both groups, directing the kicks toward the target and improving the infants' performance in the task through repetition, however, the infants with Down syndrome had lower frequencies of kicks.

## Introduction

Down syndrome is caused by a trisomy of chromosome 21, with an incidence of approximately 1/700 live births^1^, and it is associated with various complex clinical phenotypes, such as a smaller size of the cerebellum and the temporal and frontal lobes^1^ ^,^ ^2^. Some neuromotor changes are also observed, such as muscular hypotonia, joint hyperextensibility, and disturbance in the postural control and balance mechanism, which restrict the proper execution of voluntary movements^3^ ^-^ ^5^. Moreover, infants with Down syndrome may have cognitive deficits which can influence the performance of motor tasks^6^. These characteristics or organic changes can lead to a delay in the acquisition of motor skills such as reaching, sitting, and kicking, which are precursors to the development of more complex skills such as crawling and walking^7^ ^-^ ^9^.

The kick is one of the earliest motor behaviors, being observed from intrauterine life^10^. It is characterized by the cycle of flexion movement of the joints of one or both lower limbs, followed by extension and flexion again^11^ ^,^ ^12^. Between the age of 1 and 4 months, there is a marked decline in the number of alternating leg movements followed by a period in which there is the emergence of unilateral kicking and, at about 4-5 months, the new patterns of bilateral coordination become more prominent^13^. Moreover, around 5 months, the infants prefer to reach to explore the environment, reducing the frequency of kicks^14^. Thus, throughout the development of this ability, infants present in-phase and out-of-phase movements and intra- and interlimb coordination, which favors increased strength and limb coordination^13^. Thus, with a more developed movement pattern, the infant is able to perform complex tasks such as crawling and walking^10^ ^,^ ^15^ ^-^ ^18^.

The movement of kicking as a motor ability is influenced by the interaction between elements of the organism, environment, and task specificity^18^ ^-^ ^20^. In order to verify the influence of factors extrinsic to the organism (mobile reinforcement and additional weight), some authors^15^ examined the movement of kicking in healthy 4-month-olds by using a reinforcement task of kicking a touch pad which activated a mobile and adding an ankle weight of 5% the total mass of the limb to the lower limbs. For this, the authors designed the following experimental protocol: 2 minutes of baseline (spontaneous kicking), 8 minutes of acquisition (mobile reinforcement with weight), and 4 minutes of extinction (spontaneous kicking). The authors observed that the variable frequency of contact, which indicates if the infant is able to learn the task, increased during acquisition. This finding suggested that 4-month-olds could effectively accomplish the 'mobile with weight' task and these infants displayed a significant learning effect in the acquisition condition.

Another study^21^examined the influence of factors extrinsic to the organism, but in children with Down syndrome with corrected chronological age ranging from 4 to 6 months compared with typically developing children matched for chronological age and motor age, defined by the psychomotor items of the Bayley Scales of Infant Development. For this, the infants were positioned in supine and their lower limb movements were filmed for 8 minutes in four experimental conditions: control (no stimulus), verbal (verbal stimulus with caregiver), mobile (with visual stimulus of the mobile), and enriched (with verbal stimuli and touch from the caregiver). The authors found no difference between groups in the frequency of lower limb movements in any of the experimental conditions, however the infants demonstrated fewer of the more complex movements. Moreover, the authors found differences in frequency between the conditions of movement, with an increase of frequency of limb movements in the verbal condition when compared with control condition, followed by a decrease in the mobile condition, and subsequently an increase in the enriched condition. Thus, the authors concluded that the context influences the frequency of lower limb movements.

Given the above, it appears that the gradual increase in weight acts as an important stimulus for muscle strengthening and a more coordinated movement of the lower limbs, in addition to being highly correlated with the time of onset of gait, as observed in healthy infants^18^ ^,^ ^20^. Furthermore, these authors verify the additional influence of weight on the frequency of kicks because the additional weight alters both the environmental (gravitational torque) and organismic (moment of inertia) context of constraints^15^ ^,^ ^18^.

It has already been demonstrated that factors extrinsic to the organism (mobile and verbal stimulus) influence the behavior of kick in infants with Down syndrome, however, there are no studies that have investigated the effect of additional weight in these infants. This gap in the literature justifies this work, in which the results will provide a better understanding of the motor development of infants with Down syndrome and support for physical therapy treatment in this population, favoring a more complex pattern of movement and influencing the time of onset of gait. Therefore, this study aims to analyze and compare the variables frequency of kicks, frequency of foot contact with the touch pad, and frequency of success in raising the pad with and without additional weight in 3 and 4-month-old infants with Down syndrome and typical infants.

To this end, the following hypotheses were tested: 1) the variables frequency of kicks, frequency of foot contact with the touch pad, and frequency of success in raising the touch pad in infants with Down syndrome will be significantly lower when compared to typical infants due to organic changes such as hypotonia and muscle weakness; 2) the variables frequency of kicks, frequency of foot contact with the touch pad, and frequency of success in raising the pad will not be significantly different between 3 and 4 months of age for the group with Down syndrome due to delayed motor development; 3) these variables will be significantly lower at four months of age for the group of typical infants because it is the acquisition phase of reaching and grasping movements when infants prefer to explore the environment with their hands and the frequency of kicks decreases; 4) the variables frequency of kicks, frequency of foot contact with the touch pad, and frequency of success in raising the touch pad will be significantly lower in the weight experimental condition compared to the baseline and post-weight experimental conditions due to the greater difficulty of the task; and 5) these variables will be higher in the post-weight experimental condition when compared to the baseline experimental condition because it favors learning and a more complex kick pattern.

## Method

This experimental and longitudinal study was approved by the Human Research Ethics Committee of Universidade Federal de São Carlos (UFSCar), São Carlos, SP, Brazil (Process No. 081/2006). The subjects were selected from two Basic Health Units of São Carlos, and the parents signed a consent form agreeing to the participation of the infants in the study, which included a convenience sample.

### Participants

The study included 5 infants with Down syndrome and 5 infants with typical development (Table 1). The infants did not play with mobiles at home, but only with plush toys held by their caregivers. The infants with Down syndrome were not participating in any therapy programs. The typically developing group was matched for gender and chronological age with the Down syndrome (DS) group.

**Table 1.** Demographic data of patients (n=10).

		Down Syndrome	Typical	p-value
**Number of subjects **(male/female)		5 (3/2)	5(3/2)	**----**
**Gestational age **(week)		36.60 (±0.45)	37.40 (±0.54)	0.05
**Birth Weight **(Kg)		2.11 (±0.12)	2.70 (±0.32)	0.013*
**Birth Height **(cm)		42.14 (±0.56)	43.02 (±0.64)	0.65
**Apgar**	**1** ^st^ ****	8.6 (±0.45)	9.0 (±0.00)	0.17
	**5** ^th^ ****	9.4 (±0.53)	9.6 (±0.44)	0.58

* The differences are tested using the t-test for independent samples and found significant differences between groups for the variable weight

Infants with orthopedic or sensory changes, hearing loss or cardiac complications were excluded. The diagnosis of DS was confirmed by the medical report of cytogenetic analysis. Subject participation was interrupted if they were absent from any of the two assessments, if they showed events that could influence the results or when their parents did not want to continue.

### Procedures

The children were assessed on their 3 and 4 month birthday, considering a range of approximately ±5 days. At the Laboratory, the infants were undressed by their mother and the examiner collected the anthropometric measurements (weight and total body length, length, and circumference of thighs and legs, length and width of feet) to estimate the mass of each lower limb^22^.

After these procedures, infants were positioned in supine on a table developed for the study and stabilized by their shoulders by a research assistant. At the lower end of the table, there was a touch pad that when lifted by the infant's feet, activated a mobile set at a height of approximately 30 cm above the infant's face, acting as visual and auditory stimulus for the performance of kicks^12^. The touch pad position was defined by of multiplication the length of the infant's lower limb by sine 30°^15^.

**Figure 1.**
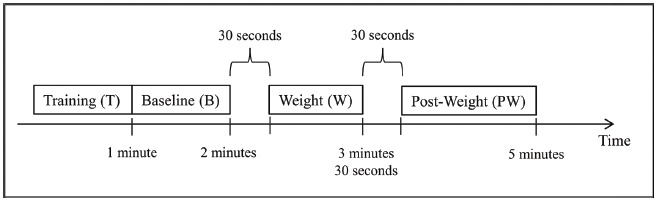
Temporal representation of experimental conditions.

The experiment was divided into four conditions: 1) training (T) - the infant's feet were placed by the examiner on the touch pad to activate the mobile, being performed three times with each limb separately and three times with both limbs together; 2) baseline (BL) - infants were free to perform kicks and could raise the touch pad with their feet and activate the mobile; 3) weight (W) - identical to the previous condition, however the infant had an ankle weight corresponding to 1/3 the weight of the lower limb; 4) post-weight (PW) - same as BL. In each condition, 1 minute was allowed for the performance of kicks and a 30-second interval was given between conditions 2, 3, and 4 for the placement or removal of the ankle weight. The entire test lasted about 5 minutes (Figure 1). Throughout the procedure, the infants remained in an active, alert state^23^.

To record the assessments, two JVC digital cameras were used (Model GY DV-300) mounted on tripods positioned anterolaterally to the chair, one on each side, with two light sources placed next to the cameras and directed towards the wall. After the recordings, an examiner placed a black bar over the infants' faces to conceal their identity. After viewing the infants in the video, another examiner counted the frequencies of motions according to the variables defined below. This count was performed by a single examiner.

### Variables analyzed

Kicking movement was defined as the movement of one or both lower limbs, starting from full flexion of the hip, knee, and ankle to extension and returning to the starting position, thus indicating a kicking cycle^11^ ^,^ ^12^. From this definition, the following variables were evaluated: 1) frequency of kicks, which corresponds to the total number of kicks performed by the infant in each experimental condition; 2) frequency of foot contact with the touch pad, which represents the number of kicks with one or both feet (simultaneously or not) directed towards the touch pad that made contact with it; 3) frequency of success to raise the touch pad, which occurred when one or both feet (simultaneously or not) made contact with the touch pad, raising it and activating the mobile^12^ ^,^ ^23^.

### Statistical analysis

The Shapiro-Wilk normality test and Levene's test for homogeneity of variance were performed. Then, three-way MANOVA (group, age, and condition) followed by Tukey's post hoc was performed for each dependent variable (frequency of kicks, frequency of foot contact with the touch pad, and frequency of success in raising the touch pad). All p-values <0.05 were considered significant. Data were organized and tabulated using the Statistical Package for the Social Sciences (SPSS 17).

Following the above-mentioned analysis, we performed a power calculation, resulting in a power of 98% for the variable frequency of success in raising the touch pad in infants with DS when comparing the baseline and post-weight conditions at 3 months (BL - mean 2.2±0.84 and PW - mean 5.0±0.71) and a power of 97.6% at 4 months (BL - mean 2.6±1.14 and PW - mean 7.2±1.48).

## Results

**Figure 2.**
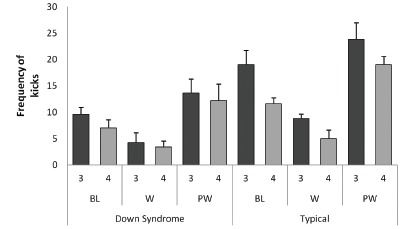
Frequency of kicks of infants with Down syndrome and typical infants at 3 and 4 months of age in the baseline (BL), weight (W), and post-weight (PW) conditions.

**Figure 3.**
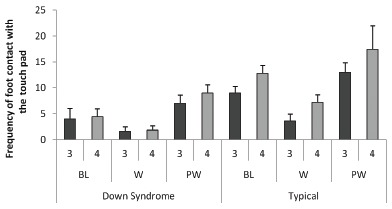
Frequency of foot contact with the touch pad in infants with Down syndrome and typical infants at 3 and 4 months of age in the baseline (BL), weight (W), and post-weight (PW) conditions.

**Figure 4.**
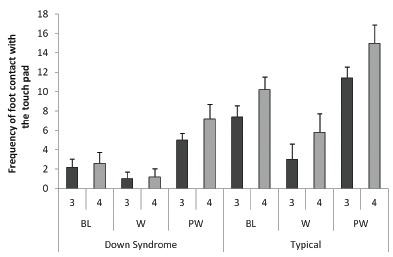
Frequency of success of infants with Down syndrome and typical infants at 3 and 4 months of age in raising the touch pad in the baseline (BL), weight (W), and post-weight (PW) conditions.

Significant differences were found when comparing groups for the variables frequency of kicks [F=1.08, p=0.011], frequency of foot contact with the touch pad, [F=6.63, p=0.013] and frequency of success in raising the touch pad [F=13.32, p=0.001], with DS infants showing a lower frequency when compared to typical infants (Figures 2, 3 and 4). There was no significant difference between ages [F=1.73, p=0.195] in the DS group for the variables frequency of kicks [F=2.05, p=0.159], frequency of foot contact with the touch pad [F=6.63, p=0.013], and frequency of success in raising the touch pad [F=0.17, p=0.679] (Figure 1B). However, for the group of typical infants, there were differences between ages for frequency of kicks [F=6.18, p=0.001], frequency of foot contact with the touch pad [F=4.45, p=0.001], and frequency of success in raising the touch pad [F=3.76, p=0.001], with 3-month-olds showing a higher frequency of kicks, except in the experimental weight condition (Figures 2, 3 and 4).

In the latter condition, the frequency of kicks was lower (p<0.001) when compared with the post-weight condition in both groups and ages. Additionally, the frequency of foot contact with the touch pad and the frequency of success in raising the touch pad was lower in the weight condition when compared to the baseline (p<0.001) and post-weight (p<0.001) conditions. Furthermore, the frequency of contact and frequency of success in raising the touch pad in the baseline condition was lower when compared to the post-weight condition (p<0.001; Figures 2, 3 and 4).

## Discussion

The present study analyzed and compared the frequency of kicks with and without additional weight in infants with DS and infants with typical development at 3 and 4 months of age. We found that infants with DS showed lower frequencies when compared to the group of typical infants in the variables frequency of kicks, frequency of foot contact with the touch pad, and frequency of success in raising the touch pad, confirming the first hypothesis. This result can be explained by the high incidence of muscle coactivation^24^, hypotonia^25^, and ligament laxity^21^. These characteristics hinder the transmission of contractile forces to the bone structures^26^ and the ability to maintain concentric and eccentric contraction of the leg muscles against gravity during the task^25^. Thus, these factors restrict the movements of infants with DS and make it difficult for them to challenge gravity and explore the environment. Furthermore, these infants can have cognitive deficits that affect the execution of motor tasks due to comprehension difficulties^6^.

Given these characteristics and their consequences, infants with DS have a delay in the acquisition of motor skills and a slower development of these skills; however, it is noteworthy that the development of these infants occurs in the same sequence as typical infants^7^ ^,^ ^9^ ^,^ ^21^. Thus, the present study confirms previous studies when confirming the second hypothesis, namely, that there was no significant difference between DS infants at 3 and 4 months of age in the variables frequency of kicks, frequency of foot contact with the touch pad, and frequency of success in raising the touch pad.

For the group of typical infants, significant differences were observed between ages 3 and 4 months in the variables frequency of kicks, frequency of foot contact with the touch pad, and frequency of success in raising the touch pad. It was observed that in the baseline and post-weight conditions, these variables were lower at 4 months of age because between 3 and 6 months of age, infants begin to perform reaching movements, exploring the external environment with their upper limbs^16^ ^,^ ^27^ and reducing the frequency of kicks^23^. However, it was observed in the weight condition that 3-month-olds had a lower overall frequency of kicks compared to 4-month-olds because the former have less muscle strength and the additional weight made the performance of the movement and task more difficult^23^. These results refute the third hypothesis.

Comparing the experimental conditions, there was a decrease in the values of all variables in the weight condition for both groups at 3 and 4 months of age, confirming the fourth hypothesis. This variation may have occurred due to the adaptation of the infants to the ankle weights. Additional weight is an environmental and organismic constraint in the task context because it makes the task more difficult, forcing the infant to produce greater muscular force to overcome gravity and hindering the performance of the kicking movement thus reducing all frequencies^15^. However, when this constraint is maintained for a period time, neural plasticity can occur, resulting in improved performance and/or new behavior^11^. In other words, practice combined with feedback (in this case, the mobile and the additional weight) causes a momentary change in the performance of the task and hence the frequency of kicks. These momentary changes are reflections of neural activations that, with practice, cause neural plasticity^28^. Thus, the infant undergoes a change in motor behavior due to neural activation and alteration^18^.

By comparing the baseline and post-weight experimental conditions for the variables frequency of foot contact with the touch pad, and frequency of success in raising the touch pad, it was found that infants from both groups increased the frequency of these variables in the post-weight condition. These results show that the additional weight had a positive influence on the kicking movement, providing proprioceptive and tactile information that acts as sensory cues during the performance of kicks, and after weight removal, these cues facilitate the movement, leading to an increased frequency of contact^29^and success. It is noteworthy that adding weight to the lower limb also increases neural activation and improves movement patterns^18^, acting as a stimulus that activates muscle proprioceptors, leading to muscle activation and movement of the lower limbs. Moreover, weight activates mechanoreceptors that perceive it and use this information to select an adaptive pattern of the kicking movement, making it more directed towards the target^23^. In other words, the additional weight activates the proprioceptive and tactile receptors, sending the somatosensory information to the central nervous system with the information about the positioning of the limb, the muscle strength produced, and the presence of limb movement. Based on this information, the infant makes adjustments in muscle activation and joint positioning in order to perform the task in the most appropriate manner^30^. Thus, these two mechanisms justify the increased frequency of success and contact after the weight removal in both groups. However, these results refute the fifth hypothesis because there was only an increase in the post-weight frequency of contact and success.

The added weight acts as an important stimulus for both groups, especially for infants with DS, improving the movement pattern by increasing the frequency of contact and success after weight removal. In addition to improving the infant's performance in the task, the repetition of the kicking movement combined with the ankle weight can lead to an increase in the control of movement and muscular strength and resistance required for the acquisition of new motor milestones such as gait and can also influence motor learning, and these aspects are important in clinical practice.

## Limitations

The limitations of this study were the reduced number of participants and the convenience sample, which requires caution in generalizing the results to the entire population of infants with DS. Thus, the results may be considered preliminary findings. Furthermore further studies with a larger number of participants are needed even though the study showed increased power for its main variable.

## Conclusion

Thus, it could be concluded that infants with DS have delayed development of the kicking movement when compared with typical infants and that weight acts as an important stimulus for both groups, especially for infants with DS, improving the movement pattern by increasing the frequency of contact and success after weight removal.
